# Unique Genotypic Differences Discovered among Indigenous Bangladeshi Rice Landraces

**DOI:** 10.1155/2014/210328

**Published:** 2014-09-14

**Authors:** Nusrat Yesmin, Sabrina M. Elias, Md. Sazzadur Rahman, Taslima Haque, A. K. M. Mahbub Hasan, Zeba I. Seraj

**Affiliations:** Plant Biotechnology Laboratory, Department of Biochemistry and Molecular Biology, University of Dhaka, Dhaka 1000, Bangladesh

## Abstract

Bangladesh is a reservoir of diverse rice germplasm and is home to many landraces with unique, important traits. Molecular characterization of these landraces is of value for their identification, preservation, and potential use in breeding programs. Thirty-eight rice landraces from different regions of Bangladesh including some high yielding BRRI varieties were analyzed by 34 polymorphic microsatellite markers yielding a total of 258 reproducible alleles. The analysis could locate 34 unique identifiers for 21 genotypes, making the latter potentially amenable to identity verification. An identity map for these genotypes was constructed with all the 12 chromosomes of the rice genome. Polymorphism information content (PIC) scores of the 34 SSR markers were 0.098 to 0.89 where on average 7.5 alleles were observed. A dendogram constructed using UPGMA clustered the varieties into two major groups and five subgroups. In some cases, the clustering matched with properties like aromaticity, stickiness, salt tolerance, and photoperiod insensitivity. The results will help breeders to work towards the proper utilization of these landraces for parental selection and linkage map construction for discovery of useful alleles.

## 1. Introduction

Genetic diversity of crop plants is the key resource for maintaining agricultural productivity. This wealth of genetic diversity has been utilized and preserved during the natural process of domestication and cultivation of crop plants. But thousands of allelic variations of traits of economic significance remain unutilized, many of which are found in traditional cultivars or landraces. Developmental activities and increasing land-use are gradually destroying natural habitats resulting in the reduction of this diversity. The situation is further aggravated by introduction of modern varieties to replace these landraces, which has happened in the case of rice cultivars. Hence it is important to understand the evolution and ecology of rice landraces in order to catalogue and preserve them for future use.

Bangladesh is an agroeconomy based country lying astride the tropic of cancer between 20°25′ and 26°38′N latitudes and 88°01′ and 92°40′E longitudes which conserves a broad range of agroecological environmental diversity in climate, physiography, soil, and hydrology [1]. This has facilitated the development of endemic landraces, particularly in rice with distinct morphological and genetic properties. For example, adaptation has occurred to different abiotic stress environments like salinity and drought and different physiological properties like aromaticity, stickiness, or resistance to pests, and so forth. These landraces have evolved and adapted according to the native environment. Most of these rice cultivars are popular among the farmers but are not high yielding enough to meet the total dietary consumption needs of the population. The diversity is also reflected in the range of rice groups cultivated in the country in different seasons like Aus (April–August), T (transplanted)-aman (July–November), B (Broadcast)-aman (March–August, and Boro (November–May) as well as in wild and weedy races. It has been reported that the IRRI Gene Bank contains more than 8,000 traditional rice varieties collected from Bangladesh [2] and many more remain to be discovered. The country lies within the origin of one of the rice domestication hubs near the Ganges valley [3] making her a reservoir of diverse rice germplasm and a crucial target for rice evolution and domestication study.

The agroecological condition in northeast region of Bangladesh is more diverse than the rest and provides suitable environment for growth of rice varieties with wide genetic variation. Traditional varieties still continue to be grown in large areas of the region due to their adaptation to the local prevailing conditions. Consumers do not hesitate to pay higher prices for the fine aromatic or glutinous rice found in these areas, each sought after due to unique cultural traditions. The Sundarban forest area spans one-third of the southern portion of 3 districts in Bangladesh and is known to be highly saline. Salinity levels gradually decline from west to east [4]. The popular landraces of this region are therefore well adapted to the soil conditions including soil salinity and are therefore regarded as possessing some resistance to salt stress, particularly at the seedling stage [5]. Landraces adapted to these stressed environments often possess some unique genes which enable them to survive. Discovery of a unique protein kinase Pstol1 that enhances root growth to survive phosphorous deficient, drought-prone soils in the traditional aus cultivar Kasalath [6] and the submergence tolerance gene* SUB1A *in another aus variety FR13A [7] and their absence in the reference rice genome establishes the importance of conserving and exploiting traditional germplasm. Despite the introduction of modern varieties, many farmers still prefer the traditional landraces in many areas. The special tolerance/beneficial characteristics of the traditional rice can therefore be considered as donor traits for introgression into high yielding rice for varietal development. Moreover, there is a need to widen the genetic base of existing salt tolerant commercial varieties using these cultivars as donors because of their adaptability to different agroecological conditions common in most coastal areas. Rice being the staple food of Bangladesh constitutes more than 70% of the total calorie intake and accounts for nearly 18% of the gross domestic product (GDP). In order to feed the increasing population in the limited land area available for cultivation in Bangladesh, the need to increase rice production is urgent. Stress tolerant high yielding varieties, therefore, need to be developed. Adapted rice landraces and their diversity analysis can therefore be an important source of information for breeding programs geared to increasing rice productivity.

Genetic uniqueness is brought about by two factors, inheritance and new mutations or deletions. Since all genetic differences between individuals are present in the primary sequence of their genomic DNA, the most straightforward method of locating uniqueness is by identifying a variant sequence in an individual for the genome under study [8]. Uniqueness is also related to biodiversity and adaptation and is modified by natural selection. DNA markers depict genome sequence composition, thus enabling the detection of differences in the genetic information carried by the different individuals. Microsatellites are PCR-based markers that can detect a significantly higher degree of polymorphism in rice [9, 10] which is therefore ideal for studies on genetic diversity and extensive genetic mapping [11]. Rice microsatellite markers have been found to be of particular value in accurate analysis of closely related rice genotypes compared to other markers [12, 13]. Availability of next generation sequencing-based genotyping methods cannot decrease the importance of microsatellite markers for diversity analysis and fingerprinting if information content, cost, and simplicity are considered.

Since polymorphic microsatellite markers show different banding pattern among varieties, those which show distinctive banding pattern can be used as a unique identifier for that specific variety. The divergent germplasm collection at IRRI and BRRI has the problem of duplication of name since many traditional varieties are cultivated in multiple regions of the country and are known by different names due to variation in local languages. This underlines the importance of unique identifiers for determining the singularity of these landraces.

In the present study, 38 different varieties of rice from different regions of the country were used for fingerprinting. The study included some recently released stress tolerant rice varieties. The objective of the study was to predict unique identifiers that can be used to determine the identity of any specific variety for further studies. An identity map using the GGT software has also been produced. Assessment of this genetic variation and grouping through similarity and dissimilarity will be valuable for proper selection of parents in breeding and mapping for introgression of novel traits from rice landraces in order to develop improved varieties. Thus the information generated from the study will be used in identifying efficient strategies for the sustainable management of the genetic resources of rice crops in Bangladesh.

## 2. Methods

### 2.1. Plant Materials

**Figure 1:**
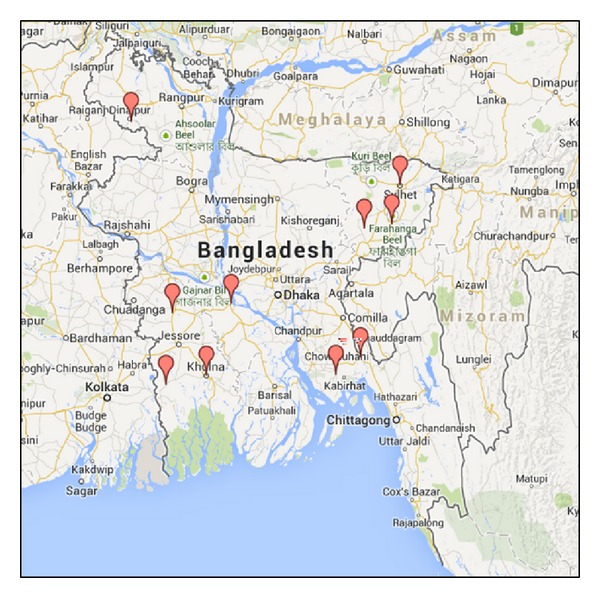
Collection sites for rice samples.

A total of 38 cultivars with different characteristics like aromaticity (Kataribhog, Chinigura), stickiness (Beruin varieties), and salt tolerance (Pokkali, Horkuch, and Boilam) as well as farmer popular landraces of some regions along with BRRI derived modern varieties BRRI dhan41, BRRI dan53, BRRI dhan55, and BRRI dhan56 were subjected to DNA fingerprinting. The varieties were collected from different regions of Bangladesh (Table 1, Figure 1). Details of the mode of seed collection from farmers can be found in previous studies [5, 14]. Rice (*O. sativa* L.) landraces grown in the coastal areas were also obtained from the gene bank at the Bangladesh Rice Research Institute (BRRI). These lines were collected in 1973 and were assigned accession numbers when entered into the gene bank. The IRIS accessions along with available phenotypic information have been included in Table 1. The collected seeds were germinated to produce plants in a net-house at Dhaka University.

### 2.2. Microsatellite Genotyping

Genomic DNA was isolated from (0.5–1.0 g) pooled leaf tissue using the modified CTAB method [15] and was quantified using the nanodrop spectrophotometer (nanodrop 1000). The quality of the DNA was assured by checking in 0.8% agarose gel in TAE buffer. A total of 34 pairs of SSR primers were used to amplify DNA from the leaves. The distribution of the selected SSR primers was even throughout the 12 rice chromosomes (see Supplementary 1 in Supplementary Material available online at http://dx.doi.org/10.1155/2014/210328). The 34 markers were selected on the basis of the genome-wide distribution from a previous list of highly polymorphic 83 markers identified in earlier studies on different rice landraces (Z. I. Seraj et al., unpublished data). Chromosomal location, SSR-motifs, annealing temperatures, and amplified product size ranges are summarized in Supplementary 2.

Polymerase chain reaction (PCR) was performed using 50 ng template DNA, 0.1 mM dNTPs, 0.33 *µ*M of forward and reverse primers each, 1.6 mM MgCl2, 1x PCR buffer, 2.6% DMSO, and 0.5 unit of recombinant Taq polymerase (Invitrogen). The mixture was then denatured at 95°C for 5 mins, followed by 35 cycles of denaturation at 95°C for 1 min, 1 min of annealing at 55-60°C (depending on primer's Tm), 1 min of extension at 72°C with a final extension step at 72°C for 7 min. The 34 RM markers were obtained commercially from idt-1st base, Singapore. The amplified PCR products were resolved and visualized in nondenaturing 10% polyacrylamide gel electrophoresis. The gels were stained in EtBr and visualized with alpha imager gel documentation system.

### 2.3. Scoring for Polymorphic Bands

For the microsatellite DNA fingerprinting of the cultivars, polymorphism was scored according to their molecular weight on polyacrylamide gels by the “molecular weight analysis” Alpha Ease FC imaging system (http://www.alphaimager.com/). The genotyping was done using the Powermarker Software [16]. The selected dataset is genotype data, the “unknown” is gametic phase for the data type, and the missing numeric is −9/−9.

### 2.4. Analysis of Unique Identifier

GGT2 [17] was used to locate the unique identifiers. Different alleles for each marker were denoted as different letters to incorporate into the GGT2 software. Distinctive banding patterns among the varieties were noted.

### 2.5. Cluster Analysis

Cluster analysis was based on similarity matrices using the unweighted pair group method with arithmetic mean (UPGMA) [18] and the relationship between cultivars was visualized as a dendrogram using Powermarker and MEGA5. The UPGMA tree was constructed by using the frequency-based distance for the “shared allele” [19].

## 3. Results 

### 3.1. Allele Frequency

**Figure 2:**
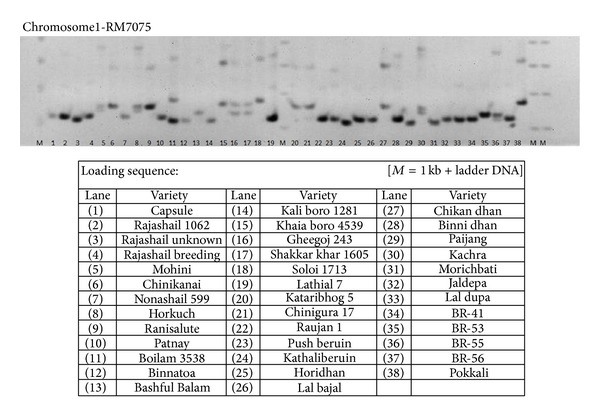
Representative gel showing the polymorphic bands between the varieties. (genotyped with RM7075).

Using 34 rice microsatellite (RM) markers, a total of 258 reproducible polymorphic bands or alleles were identified from 38 rice landraces. On average, 2–13 polymorphic alleles were found (ranging from 75 to 450 bp) across the various rice genotypes. The allelic pattern of landraces using SSR primers representing the 12 rice chromosomes is shown in Figure 2 and Supplementary 2. Most alleles were similar to the reported range of size (International Rice Genome Sequencing Project 2005) (Supplementary 2). For the microsatellite DNA fingerprinting, polymorphic bands on polyacrylamide gels were scored according to their molecular weight with reference to the Ladder marker. Major allele frequencies, allele number, and gene diversity from the gel scores were calculated by cluster analysis (Table 2).

### 3.2. Polymorphism Information Content (PIC)

Polymorphism information content (PIC) was estimated for each of the 34 markers by Powermarker Software using the equation by Botstein and coworkers [20]. Higher value of PIC score indicated higher polymorphism of the SSR marker and therefore helped to select the best SSR markers in phylogenic analysis. The PIC values obtained for the 34 markers from Powermarker [16] are listed in Table 2. Highest PIC value (0.8936) was observed for RM440 which has 13 alleles among the 38 varieties. Also RM180, RM261, RM3412, and RM27933 have very high PIC scores and high number of alleles.

### 3.3. Unique Identifier

**Figure 3:**
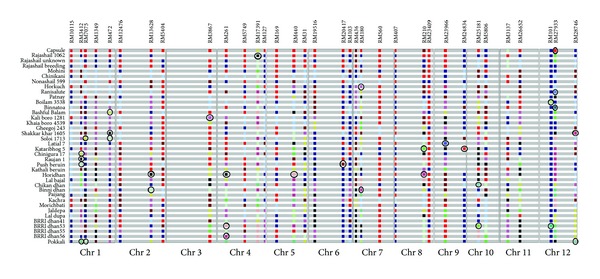
Identity map for all varieties created using GGT software [17] showing the unique identifiers among 12 chromosomes of rice. (*The unique identifiers are circled).*

Analysis of 34 polymorphic SSR markers generated a total of 34 unique bands on PAGE for 21 cultivars. Alleles were represented by different color codes in the GGT Software. Any unique color in the location of a specific marker represents the unique allele or banding of that SSR marker. The unique identifiers observed in the current study are well distributed among all chromosomes of rice except chromosome 6 and 11. No unique identifiers could be located in these 2 chromosomes in the current study. Most of the unique identifiers found in this study have high PIC score except RM 101 which had the very low PIC score of 0.099. It is however unique for the varieties Boilam 3538, BRRI dhan53, and Nonashail 599. The unique identifiers represented by GGT are given in Figure 3 (circled) and all are listed in Table 3. The SSR markers, RM 3412, followed by RM 472, RM 261, RM27933, and RM 28746 which also had high PIC were shown to provide unique identity to a number of cultivars (Figure 3).

### 3.4. Cluster Analysis

**Figure 4:**
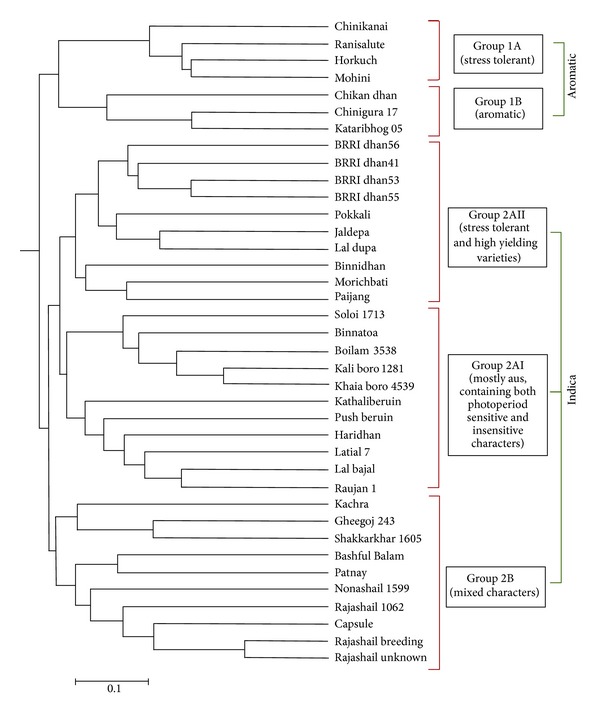
Dendogram constructed based on the polymorphism of the 38 varieties using the 34 markers used in the study.

From the dendrogram (Figure 4) two major parts of the tree were observed, groups 1 and 2. The first group is further subdivided into 1A and 1B. The 2nd group is subdivided into 2A and 2B. However, group 2A was split into two branches 2AI and 2AII. Horkuch, Ranisalute, Chinikanai, and Mohini are under the same clade in 1A. Horkuch and Ranisalute are local salt tolerant varieties, whereas Chinikanai is an abiotic stress-tolerant aromatic variety. Ranisalute was also identified as an aromatic variety in a previous study [21]. All 3 of them were also found in the aromatic clade in a diversity analysis study for salinity tolerance, conducted with 384-plex indica/indica SNP arrays which could distinguish subgroups within indica germplasm [22]. Mohini also known as BR15 is moderately tolerant to rice hispa (braconid wasp), that is, a biotic stress tolerant variety with medium and fine grain [23]. The popular aromatic varieties like Kataribhog, Chinigura 17, and Chikandhan are clustered together in 1B. Besides Mohini all other cultivars which fall in clade 1 have been previously reported in an aromatic subgroup which indicates that the two major groups found in the current study can be annotated as aromatic (group I) and indica (group 2). Modern BRRI derived moderately abiotic stress tolerant varieties like BRRI dhan41, BRRI dhan53, BRRI dhan55, and BRRI dhan56 are grouped under the same category in group 2AII. The well-known salt tolerant cultivar Pokkali also belongs to this group. Although the modern varieties are stress-tolerant, they are diverse from the traditional stress tolerant genotypes in 1A. Soloi 1713, Kaliboro 1281, khaiaboro 4539, Boilam 3538, and Binnatoa are clustered together within the larger subgroup, 2AI. These are Aus and photoperiod insensitive landraces from the mildly saline coast of the mid northeast Noakhali, as well as nonsaline Faridpur and Habigonj. This group 2AI also contains photosensitive and glutinous varieties, but which clustered separately. Patnay, BashfulBalam, Nonashail599, Rajashail varieties and Capsule are from the mild to moderate saline prone regions of Noakhali, Khulna, and Satkhira and are clustered together in the clade, 2B.

### 3.5. Pattern of Motif Observed among the Polymorphic SSR Markers

The markers used in this study had di-, tri-, or tetranucleotide motifs. 55.88% markers were dinucleotide, 38.26% were trinucleotide, and only 5.88% were tetranucleotide. Among the 10 highly polymorphic markers, 60% had dinucleotide motif, 30% had trinucleotide motif, and only 1% had tetranucleotide motif which shows the high polymorphism value of dinucleotide motif-containing markers among the Bangladeshi rice landraces.

## 4. Discussion

The rice germplasm in Bangladesh is vast and divided mainly into three different major groups, that is, indica, aus, and aromatic [3, 24–26]. Farmers in the region used to grow large number of local landraces with special characteristics. Many of these have already become extinct or are on their way to become so. The main reason for not planting these landraces is their low yield, long growth duration, lodging, and weak stems. So studies on genomic identification and characterization of traditional varieties are important since they possess traits which have allowed them to adapt to the native environment. Characterization of these genotypes followed by their utilization as genetic donors is crucial to maintain food security for the country in years to come. The rice collection in this study was from the southern coastal region as well as the north-east region which are well known for their saline tolerant, aromatic, and sticky varieties, respectively. Moreover, the study included recently released high yielding varieties reported to be salt and drought tolerant produced by the Bangladesh Rice Research Institute.

Genetic variations among accessions in this study were assessed using microsatellite markers. Rice microsatellite (RM) or simple sequence repeat (SSR) markers are robust and codominant (i.e., they can detect heterozygous loci), exhibit high allelic variation, and are widely distributed throughout the Oryza genome [27]. More than 1000 microsatellite markers have been characterized in rice [28] and sequences of around 20000 SSR markers are published after sequencing of the rice genome [29] which are available through a searchable interface of the Gramene database [30]. These markers are robust and are based on the variable repeat of special motifs in different varieties. DNA markers are not very easily affected by environmental factors and/or developmental stages of the plant [31, 32] making it a good choice for studying polymorphism. The unique mechanism for generation of SSR allelic diversity by replication slippage [33] is one of the reasons that SSRs show high polymorphism. Moreover, they are not limited to variations in only coding regions like allozyme loci [34]. Microsatellites are particularly important as they can be easily compared among laboratories. Ease of use, availability, and high polymorphic rate compared to the sequencing-based genotyping markers still make microsatellites the marker of choice for low cost local studies.

The molecular identity of local and BRRI varieties and their association with each other will provide important information for the better management of the rice varieties for breeding and preservation. In the current study, on average 2–13 polymorphic alleles were found (ranging from 80 to 350 bp) across the various rice genotypes. Dinucleotide repeat motifs displayed higher level of variation among the rice genotypes than the other motifs which confirmed the abundant nature of dinucleotide repeat polymorphism as reported earlier [35].

The UPGMA (unweighted pair group method with arithmetic mean) method was applied in the construction of the dendrogram that is a simple agglomerative or bottom-up data clustering method used for the creation of phylogenetic trees. UPGMA algorithm examines the structure present in a pairwise distance matrix for constructing a rooted tree or dendrogram. The fingerprinting result with 34 RM markers grouped the landraces into 2 major groups and 5 subgroups after cluster analysis. The varieties could be differentiated according to their aromaticity, stickiness properties, high yielding characteristics, photoperiodicity, and modern or traditional characteristics. Interestingly, the salt tolerant genotypes were present in 3 different groups. This suggests involvement of different sets of genes responsible for their tolerance and intercrossing could lead to a combination of different mechanisms. This hypothesis will however need to be tested further. Some varieties were grouped together even though there were no known similar properties amongst them. More markers or a different set may provide a different picture of relatedness. Major varieties in Group I constituted well known aromatic varieties like Kataribhog, Chinigura, and so forth; however, some of the varieties like Horkuch and Ranisalute are well known for their salinity tolerance but not aroma. In a recent study by Platten and coworkers [36] the association of Na+ exclusion and salt tolerance with aromatic alleles was shown based on* OsHKT* phylogeny. In another diversity study by Thomson and coworkers [22, 26] with 384 SNP markers, varieties like Horkuch and Ranisalute were also grouped with aromatic varieties as observed in the present study. These two reports along with the current findings are significant in terms of delineating markers to characterize the traditional varieties for novel traits. These also indicate that the two major branches found in the current study should be aromatic (group 1) and indica (group 2). Aromatic varieties were described as an intermediate division between indica and japonica subpopulation in some earlier studies [37], but in all current studies Aromatic varieties form a distinct subgroup from indica and japonica [3, 24, 25, 38]. Grouping of Mohini with the other aromatic varieties needs further investigation indicating some novel common property, since this is commonly known as an Aus and Boro variety with medium grain architecture [23]. In group 2A-II the BRRI derived high yielding varieties formed a well differentiated clade, while in group 2A-I some of the sticky rice varieties from northeast region grouped into the same clade.

Most of the markers were found to be of high PIC score which indicates the ability of the markers to detect polymorphism between closely related varieties in high resolution. The level of polymorphism determined by the PIC value (mean = 0.69) is consistent with the range of PIC values observed in the earlier published reports. In one of these reports the PIC values ranged from 0.24 to 0.92 with an average value of 0.61 [38], in other reports, PIC ranged from 0.19 to 0.90 with an average of 0.75 and in another study, with an average value of 0.78 [39]. The result revealed that marker RM440 which has a PIC value of 0.89 with 13 different alleles would be best in screening rice genotypes followed by RM180 and RM261.

Unique identifier can be used as a distinctive passport for local germplasm. This can be very useful in identification of variants as well as their progenies which is important for protecting the intellectual property right (IPR) for Bangladeshi accessions. A total of 34 unique identifiers for 21 varieties were observed from the analysis of 34 markers and an identity map presented (Table 3 and Figure 3). The PIC score for the unique markers ranged from 0.7 to 0.9, except RM 101 which was found to have very low PIC score (0.099). Although RM101 is less polymorphic, which has only 3 different alleles among the cultivars under study, it was interesting to note that this marker (RM101) alleles were recognized as unique identifiers for the varieties, Boilam 3538 and BRRI dhan53. In general, the unique identifiers did not show any bias in their chromosomal location except for chromosomes 6 and 11, where no unique identifier was detected for the cultivars in this study.

In short, the study could successfully locate 34 unique identifiers for 21 varieties from the analysis of 34 polymorphic SSR markers. As these markers can verify the respective varieties uniquely, they can be used in crop identity protection in breeding programs to improve rice varieties. The polymorphic markers used in the study were evaluated by PIC score which is a measure of polymorphism of the markers. This score is valuable for choosing highly polymorphic markers for linkage analysis and variety selection for breeding program of rice germplasm. A dendogram was constructed based on the frequency based distances of the 38 cultivars under study, which could cluster the cultivars in the two major subpopulation structures of* Oryza sativa* that is Aromatic Indica. This clustering could further subdivide the cultivars according to their phenotypical characteristics to some extent. This information will be useful for improvement of rice production or development of new rice varieties. Moreover, this genetic distance analysis provides the evolutionary linkage perspective between traditional and modern varieties being released in Bangladesh through international efforts of institutions like IRRI. Potential of these unique identifiers to be linked with any QTL region or special physical properties will need to be examined further. In future, inclusion of more SSR markers and cultivars from other regions of the country will be helpful in terms of characterizing the diverged rice germplasm of Bangladesh. Genotypic data obtained from these studies along with their physiological characterization can be used in construction of a database.

## Supplementary Material

Supplementary 1 depicts the distribution and location of the selected markers through the 12 chromosomes of rice. This figure shows that the markers used in the study were selected from all the chromosomes of rice. Supplementary 2 lists all the SSR markers used in the study in tabular format. This table also includes information about the primer sequences, their respective Tm, PCR product size, the SSR motif and the corresponding number of alleles and Polymorphism Information Content (PIC) value found in the current study.

## Figures and Tables

**Table 1:** List of rice varieties collected from different regions.

Variety	Accession	Location	NPGRC∗ id	Properties
Capsule	IRIS 121-7435	Satkhira, SW		Salt tolerant
Rajashail 1062	IRIS 121-7407			
Rajashail Unknown				
Rajashail Breeding				
Mohini		Khulna		Biotic stress tolerant
Chinikanai	IRIS 307-55477	Satkhira	97A00031	Fine aromatic
Nonashail 599		Noakhali		
Horkuch	IRIS 109-1513	Khulna		Salt tolerant
Ranisalute		Khulna		Salt tolerant
Patnay		Khulna	95A00208	Salt tolerant
Boilam 3538	IRIS 6-82602	Noakhali		Salt tolerant
Binnatoa	IRIS 40-171906	Noakhali		Salt tolerant
Bashful Balam	IRIS 109-1516			
Kali boro 1281	IRIS 6-94498	Faridpur		
Khaia boro 4539	IRIS 1-57186	Habiganj		
Gheegoj 243	IRIS 1-60817	Noakhali		
Shakkar khar 1605				Fine aromatic
Soloi 1713	IRIS 1-58930	Faridpur	04A02113	
Latial 7		Moulovibajar	97A00003	Also known as Lathishail
Kataribhog 5	IRIS 1-58168	Dinajpur		Aromatic
Chinigura 17		Moulovibajar		Aromatic
Raujan 1		Moulovibajar		
Push beruin		Moulovibajar		
Kathaliberuin		Moulovibajar		Semiglutinous
Horidhan		Jhenaidah		High yield
Lal bajal		Feni		
Chikan dhan				Fine aromatic
Binni dhan	IRIS 307-50894			
Paijon	IRIS 307-52869	Dinajpur		
Kachra	IRIS 6-73379	Khulna		Salt tolerant
Morichbati	IRIS 1-28568			
Jaldepa				
Lal dupa	IRIS 307-51001			
BRRI dhan41		BRRI		Mod. salt tolerant (tolerance source SR26B)
BRRI dhan53		BRRI		Mod. salt tolerant
BRRI dhan55		BRRI		Mod. salt cold tolerant (tolerance source *O. rufipogon*)
BRRI dhan56		BRRI		Mod. drought tolerant (tolerance source is unknown, probably WAY RAREM as this variety is recommended for acid soils and high active Al and Fe)
Pokkali	IRIS 121-25932	Sri Lanka		Salt tolerant

*NPGRC stands for National Plant Genetic Resource Center which contains passport data containing physiological and phenotypical properties of some of the rice included in the current study.

**Table 2:** Polymorphism information content of different SSR markers.

Marker	Major allele frequency	Allele number	Chromosome	Gene diversity	PIC
RM10115	0.39	3	1	0.6440	0.57
RM3412	0.24	12	1	0.8670	0.85
RM7075	0.36	10	1	0.8054	0.78
RM1349	0.21	9	1	0.8629	0.85
RM472	0.34	8	1	0.7576	0.72
RM12476	0.34	5	2	0.7576	0.72
RM13628	0.29	8	2	0.8130	0.79
RM5404	0.53	6	2	0.6593	0.62
RM3867	0.34	11	3	0.8186	0.80
RM261	0.21	13	4	0.8823	0.87
RM5749	0.39	5	4	0.7105	0.66
RM17391	0.29	7	4	0.7881	0.76
RM127	0.39	5	4	0.7008	0.65
RM169	0.50	3	4	0.6219	0.55
RM440	0.16	13	5	0.9017	0.89
RM31	0.24	8	5	0.8296	0.81
RM19516	0.26	7	6	0.8158	0.79
RM20417	0.47	6	6	0.6814	0.63
RM103	0.61	5	6	0.5900	0.56
RM436	0.26	7	7	0.8213	0.80
RM180	0.21	13	7	0.8906	0.88
RM560	0.79	3	7	0.3546	0.33
RM407	0.55	3	8	0.5360	0.44
RM210	0.18	11	8	0.8781	0.87
RM23409	0.79	2	8	0.3324	0.28
RM23966	0.24	9	9	0.8504	0.83
RM24834	0.66	5	9	0.5291	0.50
RM25181	0.16	10	10	0.8767	0.86
RM5806	0.18	9	10	0.8601	0.84
RM3137	0.18	10	11	0.8809	0.87
RM26652	0.21	9	11	0.8740	0.86
RM101	0.95	3	12	0.1011	0.10
RM27933	0.18	12	12	0.8795	0.87
RM28746	0.33	8	12	0.7846	0.75

**Table 3:** List of 34 unique identifiers found from the study.

Variety	Marker	Chr	PIC	MW
Capsule	RM27933	12	0.87	172
Rajashail 1062	RM17391	4	0.76	182
Chinikanai	RM261	4	0.87	110
Horkuch	RM180	7	0.88	143
	RM23966	9	0.83	226
Ranisalute	RM27933	12	0.87	160
Boilam 3538	RM101	12	0.099	319
	RM27933	12	0.87	140
Bashful Balam	RM472	1	0.72	272
Kali boro 1281	RM3867	3	0.8	216
Shakkar khar 1605	RM472	1	0.72	270
	RM28746	12	0.74	142
Soloi 1713	RM7075	1	0.77	169
	RM472	1	0.72	284
Latial 7	RM23966	9	0.83	234
Kataribhog 5	RM210	8	0.87	158
	RM24834	9	0.49	314
Chinigura 17	RM3412	1	0.85	124
Raujan 1	RM3412	1	0.85	107
Push beruin	RM3412	1	0.85	117
Horidhan	RM13628	2	0.79	215
	RM261	4	0.87	119
	RM440	5	0.89	191
	RM210	8	0.87	157
Chikan dhan	RM25181	10	0.86	137
Binni dhan	RM13628	2	0.79	268
	RM180	7	0.88	115
BRRI dhan53	RM261	4	0.87	130
	RM25181	10	0.86	158
	RM101	12	0.099	279
BRRI dhan56	RM31	5	0.81	126
Pokkali	RM3412	1	0.85	95
	RM7075	1	0.77	162
	RM28746	12	0.74	126
