# Supplementary material for: Unique Genotypic Differences Discovered among Indigenous Bangladeshi Rice Landraces
Source: Int J Genomics. 2014 Sep 14;2014:210328. doi: 10.1155/2014/210328 (PMC4180204; doi:10.1155/2014/210328)
Supplement: Supplementary file 1 — Supplementary 1 depicts the distribution and location of the selected markers through the 12 chromosomes of rice. This figure shows that the markers used in the study were selected from all the chromosomes of rice. Supplementary 2 lists all the SSR markers used in the study in tabular format. This table also includes information about the primer sequences, their respective Tm, PCR product size, the SSR motif and the corresponding number of alleles and Polymorphism Information Content (PIC) value found in the current study. [file 210328.f1.pdf]

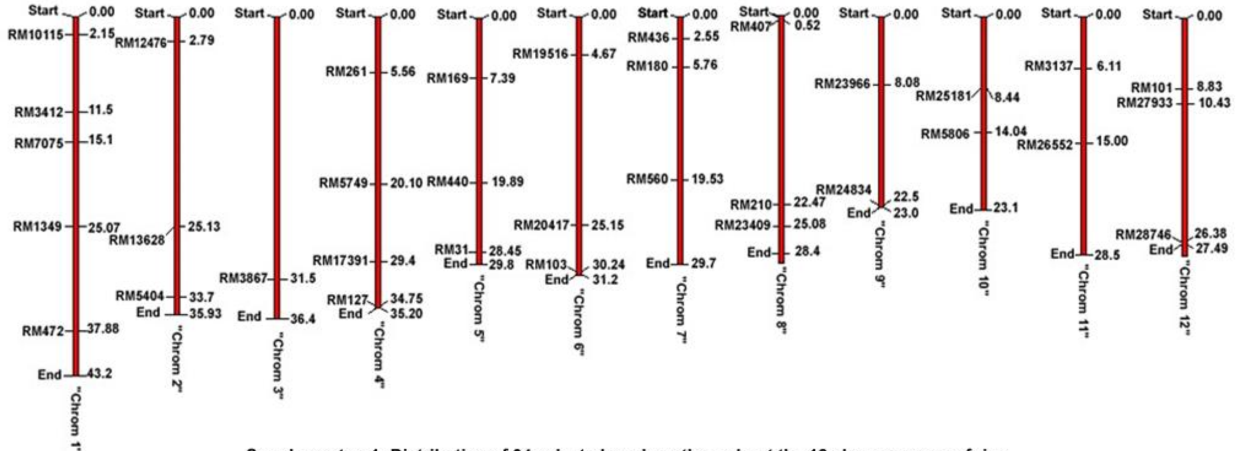


**Supplementary1: Distribution of 34 selected markers throughout the 12 chromosomes of rice**

**Supplementary 2: List of SSR markers used in the study**

| Markers | Chrom-osome | Primer sequence | | Tm | PCR Product Size (bp) | SSR Motif | Allele no. | PIC |
|---|---|---|---|---|---|---|---|---|
| | | Forward | Reverse | | | | | |
| RM10115 | 1 | acaagacgaggtaacacgcaagc | gcgaaggatcaacgatgatatgg | 63.7 | 245 | CTT | 3 | 0.5679 |
| RM3412 | 1 | aaagcaggttttcctcctcc | cccatgtgcaatgtgtcttc | 63.7 | 211 | CT | 2 | 0.8540 |
| RM7075 | 1 | tatggactggagcaaacctc | ggcacagcaccaatgtctc | 61.4 | 155 | ACAT | 4 | 0.7845 |
| RM1349 | 1 | cgttccaatattcagaacag | tttccatctcgagaagctc | 58.0 | 160 | AG | 2 | 0.8479 |
| RM472 | 1 | ccatggcctgagagagagag | Agctaaatggccatacggtg | 55.0 | 296 | GA | 2 | 0.7208 |
| RM12476 | 2 | gacgagtgatgtcagcacataaacc | actatccacagcagccattgtcg | 64.6 | 221 | TTAA | 4 | 0.7177 |
| RM13628 | 2 | tatgccacgaatgacccctaacc | Ctccatatgcagcgacaatcg | 62.6 | 282 | CT | 2 | 0.7884 |
| RM5404 | 2 | ggccatccatctcctgtatg | Gacacacacagggttggttg | 62.4 | 125 | TC | 2 | 0.6215 |
| RM3867 | 3 | ttgactggaacatcgagctc | Atcccctctacaccgtaccc | 62.8 | 120 | GA | 2 | 0.8004 |
| RM261 | 4 | ctacttctccccttgtgtcg | tgtaccatcgccaaatctcc | 55.0 | 125 | CT | 2 | 0.8714 |
| RM5749 | 4 | gtgaccacatctatatcgctcg | atggcaaggttggatcagtc | 61.5 | 162 | ACT | 3 | 0.6616 |
| RM17391 | 4 | actttgctctgaacttgcagtgg | gctagctgctatcaggattcacg | 63.7 | 175 | AG | 2 | 0.7566 |
| RM127 | 4 | gtgggatagctgcgtcgcgtcg | aggccagggtgttggcatgctg | 55.0 | 223 | AGG | 3 | 0.6497 |
| RM169 | 5 | tggctggctccgtgggtagctg | Tcccgttgccgttcatccctcc | 55.0 | 167 | GA | 2 | 0.5504 |
| RM440 | 5 | catgcaacaacgtcaccttc | Atggttggtaggcaccaaag | 55.0 | 169 | CTT | 3 | 0.8936 |
| RM31 | 5 | gatcacgatccactggagct | aagtccattactctcctccc | 55.0 | 140 | GA | 2 | 0.8079 |
| RM19516 | 6 | taaacccaaagccgccaactgc | Tcctcctcctgccctcttctcc | 66.4 | 87 | CGC | 3 | 0.7909 |
| RM20417 | 6 | gagacatcatagccggatcttcc | tcggtagaacatcacctccaagg | 64.6 | 258 | CTT | 3 | 0.6346 |
| RM103 | 6 | cttccaattcaggccggctggc | cgccacagctgaccatgcatgc | 55.0 | 336 | GAA | 3 | 0.5568 |
| RM436 | 7 | attcctgcagtaaagcacgg | cttcgtgtacctccccaaac | 55.0 | 81 | TAA | 3 | 0.7981 |
| RM180 | 7 | ctacatcggcttaggtgtagcaacacg | acttgctctacttgtggtgagggactg | 55.0 | 110 | ATT | 3 | 0.8812 |
| RM560 | 7 | gcaggaggaacagaatcagc | Agcccgtgatacggtgatag | 55.0 | 239 | CT | 2 | 0.3267 |
| RM407 | 8 | gattgaggagacgagccatc | ctttttcagatctgcgctcc | 55.0 | 172 | AG | 2 | 0.4383 |

| | | | | | | | | |
|---|---|---|---|---|---|---|---|---|
| RM210 | 8 | tcacattcggtggcattg | cgaggatggttgttcacttg | 55.0 | 140 | CT | 2 | 0.8660 |
| RM23409 | 8 | atcaggaagctgcaagaactcg | tttggagtggtccttgtactgc | 62.7 | 179 | CT | 2 | 0.2772 |
| RM23966 | 9 | caatacgtgtttcccatcgttgc | cgagttaaaccgtcgaaagatcg | 62.8 | 236 | CT | 2 | 0.8330 |
| RM24834 | 9 | tttccggccacttcctatattcc | tccagtccaatttccaagctacc | 62.8 | 350 | TA | 2 | 0.4953 |
| RM25181 | 10 | aaagagcttccctaatggcttcg | gagagaatgacctctcccaagacc | 64.5 | 162 | TTC | 3 | 0.8638 |
| RM5806 | 10 | ctaattgcggttgaagcctc | Cctcccaatctttgcacatc | 60.4 | 163 | AGG | 3 | 0.8441 |
| RM3137 | 11 | gttaggaattccatgctgcg | tgcccgtgctcgataagg | 61.3 | 197 | CA | 2 | 0.8691 |
| RM26652 | 11 | caatccattgctggttgatgc | caagatctccaaggtgctgagg | 62.6 | 169 | TTC | 3 | 0.8610 |
| RM101 | 12 | gtgaatggtcaagtgacttaggtggc | acacaacatgttccctcccatgc | 55.0 | 324 | CT | 2 | 0.0986 |
| RM27933 | 12 | tcctctgtcatatggctgtaaacg | ggacaaggaggaactattgattgg | 62.9 | 435 | TA | 2 | 0.87 |
| RM28746 | 12 | gaagaaaaagacgccaagaaacg | cattccattcccttcctcttcg | 62.8 | 157 | GAA | 3 | 0.74 |
